# Probing the ferredoxin:hydrogenase electron transfer complex by infrared difference spectroscopy†

**DOI:** 10.1039/d5sc00550g

**Published:** 2025-04-30

**Authors:** Selmihan Sahin, Johanna Brazard, Kilian Zuchan, Takuji B. M. Adachi, Ulrich Mühlenhoff, Ross D. Milton, Sven T. Stripp

**Affiliations:** a University of Geneva, Department of Inorganic and Analytical Chemistry, Sciences II Quai Ernest-Ansermet 30 Geneva 4 1211 Switzerland ross.milton@unige.ch; b Suleyman Demirel University, Department of Chemistry, Faculty of Engineering and Natural Sciences Cunur Isparta 32260 Turkiye; c University of Geneva, Department of Physical Chemistry, Sciences II Quai Ernest-Ansermet 30 Geneva 4 1211 Switzerland; d Philipps-Universität Marburg, Institute of Cytobiology, Center for Synthetic Microbiology (SYNMIKRO) Karl-von-Frisch-Str. 14 Marburg 35043 Germany; e University of Potsdam, Institute of Chemistry, Spectroscopy and Biocatalysis Karl-Liebknecht-Straße 24-25 Potsdam 14476 Germany sven.stripp@uni-potsdam.de

## Abstract

Ferredoxins are small iron–sulfur proteins that engage in one-electron transfer with oxidoreductases across all domains of life. The catalyzed reactions often include multiple electrons, *e.g.*, in the two-electron reduction of NADP^+^ during photosynthesis or the reduction of protons to H_2_ by the metalloenzyme hydrogenase. To date, the microscopic details of how ferredoxins facilitate multiple electron redox chemistry are unknown. Ferredoxins of the *Allochromatium vinosum* subfamily contain two [4Fe–4S] clusters, which allows for two one-electron transfer reactions. However, the iron–sulfur clusters of conventional 2[4Fe–4S]-type ferredoxins have very similar reduction potentials and conclusive evidence for the transfer of two electrons during a single protein–protein interaction (PPI) has not been reported. In this work, the electron transfer complexes between the clostridial 2[4Fe–4S] ferredoxin, *Cp*Fd, and [FeFe]-hydrogenases from both *Clostridium pasteurianum* (*Cp*I) and *Chlamydomonas reinhardtii* (*Cr*HydA), were investigated. Introducing a non-canonical amino acid near to one of the iron–sulfur clusters of *Cp*Fd permitted the quantification of electric field changes *via* the vibrational Stark effect by Fourier-transform infrared (FTIR) spectroscopy. Upon reduction, *in situ* FTIR difference spectroscopy reported on protein structural changes and microscale thermophoresis revealed that the affinity between ferredoxin and hydrogenase is modulated by redox-dependent PPIs. Prompted by these findings, we suggest a model how ferredoxin efficiently facilitates multiple electron redox chemistry based on individual one-electron transfer reactions.

## Abstract 2

The electron transfer complex of [FeFe]-hydrogenase and ferredoxin was probed under reducing or oxidizing conditions. Infrared spectroscopy indicates structural changes that modulate redox-dependent protein–protein interaction.

## Graphical abstract

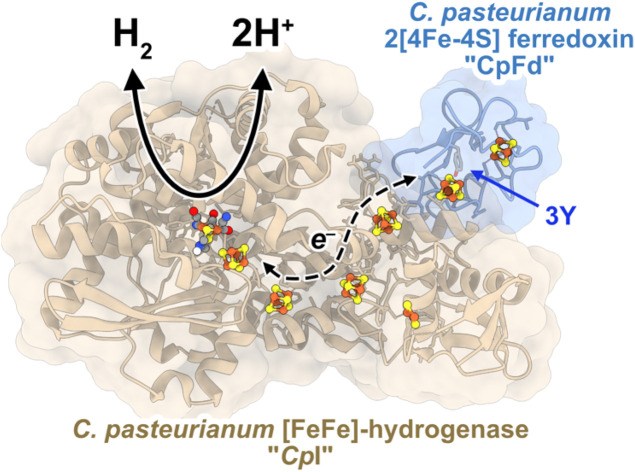

## Introduction

Protein–protein interactions (PPIs) are central to biochemical reactions,^1^ including electron transfer (ET) between metalloproteins.^2–4^ Small ET proteins such as flavodoxins, blue-copper proteins, and cytochromes^5–7^ as well as iron–sulfur proteins^8^ serve as redox partners in virtually all metabolic networks. This encompasses photosynthesis and the Calvin–Benson cycle, aerobic respiration, and the citric acid cycle, oxidative phosphorylation, and many other redox processes in the cell. Identifying PPIs is a considerable challenge as most of these interactions are transient and subtle.

**Fig. 1:**
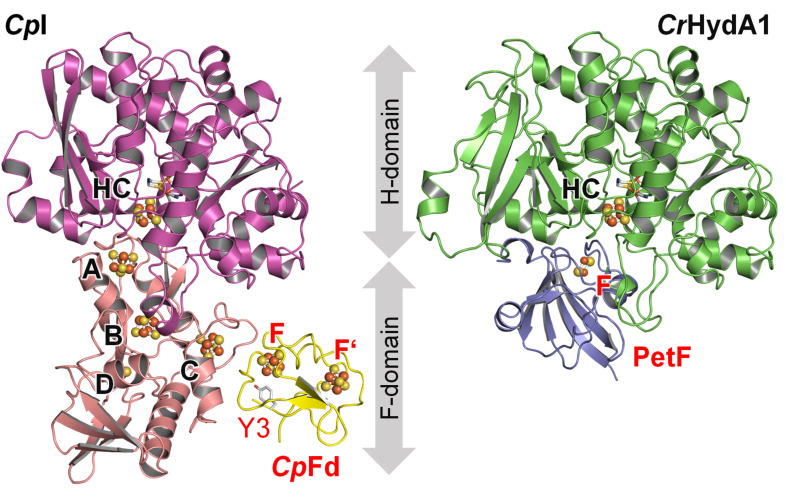
*In silico* models of the ferredoxin:hydrogenase complex. Left: [FeFe]-hydrogenase *Cp*I (PDB ID 6N59) containing the H-cluster (HC) in the H-domain (magenta) as well as [4Fe–4S] clusters A–C and [2Fe–2S] cluster D in the F-domain (pink). Ferredoxin *Cp*Fd (yellow, PDB ID 1CLF) binds two [4Fe–4S] clusters F and F′, apart by ∼9.4 Å (shortest distance). The shortest distance between clusters C and F is ∼8.0 Å. In this study, tyrosine Y3 of *Cp*Fd (highlighted) is mutated to a cyano-phenylalanine. Right: [FeFe]-hydrogenase *Cr*HydA1 (green, homology model) containing only the H-cluster (HC). An F-domain is missing. Ferredoxin PetF (blue, PDB ID 2MH7) binds a single [2Fe–2S] cluster F. The distance between clusters HC and F is ∼8.7 Å. Models generated with ClusPro 2.^33^ Coordinates for both complexes are supplied in ESI.†

Ferredoxins (Fds) are small metalloproteins that engage in ET with various oxidoreductases.^9^ They contain either [2Fe–2S] or [4Fe–4S] clusters, depending on their occurrence in eukaryotes or prokaryotes. The primary and secondary coordination sphere of ferredoxin modulates their standard reduction potential (*E*^0^′) over hundreds of millivolts,^10^ which led to the distinction of “low-potential” and “high-potential” ferredoxins.^11^ The mesophilic bacterium *Clostridium pasteurianum* produces a low-potential ferredoxin (*Cp*Fd) that contains two [4Fe–4S] clusters (*F* and *F*′, Fig. 1A) with similar reduction potentials (*E*_m_ = −400 ± 10 mV *vs.* SHE).^12^ In contrast, the 2[4Fe–4S] ferredoxin from *Azotobacter vinelandii* shows distinct *E*_m_s of −486 mV and −644 mV *vs.* SHE, as a result of two individual one-electron transfer steps at very low potential.^13^ Similar data was reported for the 2[4Fe–4S] ferredoxin from *Allochromatium vinosum*, where polar interactions with the clusters were identified to diversify the *E*^0^′s.^14^ In variance to these “alvin-type” ferredoxins, often discussed in the context of N_2_ fixation,^15^ Burgess and co-workers have argued that the homogeneity of *E*^0^′s in clostridial ferredoxins should allow for both one- and two-electron transfers at the same potential.^13^ To the best of our knowledge, however, two-electron reduction of clostridial ferredoxin has not been shown, and it is established that *Cp*Fd likely acts a one-electron carrier functioning at similar *E*^0^′s like eukaryotic ferredoxins.^16^

Ferredoxin *Cp*Fd is well-suited for ET with hydrogenase (Fig. 1). Hydrogenases are oxidoreductases that catalyze the reversible conversion of molecular hydrogen (H_2_), two protons (H^+^), and two electrons (hydrogen turnover).^17,18^ In the class of [FeFe]-hydrogenase, catalysis takes place at an unique iron–sulfur-based cofactor, the “hydrogen-forming” H-cluster, which is connected to the protein surface by a proton transfer pathway^19^ and an electron relay of various iron–sulfur clusters in the so-called F-domain.^20–22^ While the [FeFe]-hydrogenase from *C. pasteurianum* contains three [4F–4S] clusters and one [2Fe–2S] cluster (*Cp*I, Fig. 1), only two [4Fe–4S] clusters are found in the [FeFe]-hydrogenase from *Desulfovibrio desulfuricans*, and no such “accessory” clusters are found in the [FeFe]-hydrogenase from *Chlamydomonas reinhardtii* (*Cr*HydA1, Fig. 1).^23^ These enzymes are of interest to biotechnological H_2_ production, given their high proton reduction rates of up to 10 000 s^−1^ close to the formal (or, biological standard) potential of hydrogen (*E*^0^′_2H+/H_2__ = −414 mV *vs.* SHE).^24–26^*In vivo*, [FeFe]-hydrogenase competes for ferredoxin with other oxidoreductases, *e.g.*, in NAD(P)H production, lipid maturation, and N_2_ fixation.^27–29^ Understanding electron flux in hydrogen turnover, it is important to characterize intermolecular ET between [FeFe]-hydrogenase and ferredoxin (Fig. 1), which relies on PPIs and, presumably, a successive exchange of single electrons.^30–32^

Fourier-transform infrared (FTIR) spectroscopy is ideally suited to detect PPIs, mainly due to its sensitivity detecting small changes in the hydrogen-bonded C=O and N–H groups of the peptide backbone that are informative of secondary structural changes.^34^ However, the spectral overlap with solvent (H_2_O) complicates the analysis, in particular when transient and/or subtle changes are addressed. These challenges can be overcome by FTIR difference spectroscopy where the activity of a sample is induced by a specific trigger (light, potential jumps, reactant titrations, *etc.*) that allow analyzing the spectral changes rather than the absolute spectra.^35–39^

Introducing specific chromophores by protein engineering is a complementary strategy. Here, the reporter group shows favorable spectroscopic properties like a high extinction coefficient or a distinct absorption frequency. This can be achieved by amber codon suppression using an aminoacyl-tRNA synthetase/tRNA pair designed for the selective insertion of non-canonical amino acids^40^ like 4-cyano-l-phenylalanine (pCNF), which contains an IR-active nitrile group (–C≡N) that can be probed to measure changes in the electric field *via* the vibrational Stark effect (VSE).^41–43^ The VSE originates from the small differences in dipole moment between the first vibrationally exited state *ν*_1_ compared to the ground state *ν*_0_. An external electric field projected onto the nitrile group by the protein environment interacts differently with the *ν*_1_ or *ν*_0_ dipole moment.^44^ The measured frequency shift Δ*ν* is then proportional to the electric field vector 
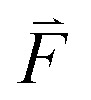
 with the negative difference in dipole moments 
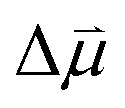
, the later which is also referred to as Stark tuning rate. The introduction of pCNF within a protein yields a local electric field probe.^45^

To investigate site-selective redox changes in ferredoxin *Cp*Fd under turnover conditions, we replaced tyrosine Y3 by the non-canonical amino acid pCNF near the [4Fe–4S] cluster F (Fig. 1). With the aim of minimizing any other changes to *Cp*Fd, we targeted the only tyrosine residue of the protein. Our *Cp*Fd:*Cp*I model indicates that the pCNF residue is positioned ∼6.0 Å from the *Cp*Fd:*Cp*I interface, and that the distal [4Fe–4S] cluster of *Cp*I (C) is about 11.4 Å away (Fig. S1†). We used *in situ* attenuated total reflectance (ATR) FTIR difference spectroscopy to investigate *Cp*Fd in complex with [FeFe]-hydrogenases *Cp*I or *Cr*HydA1, triggering reduction and oxidation by changing the gas atmosphere between H_2_, N_2_, or O_2_. Only in the *Cp*Fd:*Cp*I complex, a subtle yet significant shift of the nitrile band was observed, accompanied by clear changes in protein secondary structure.

## Results and discussion

**Fig. 2:**
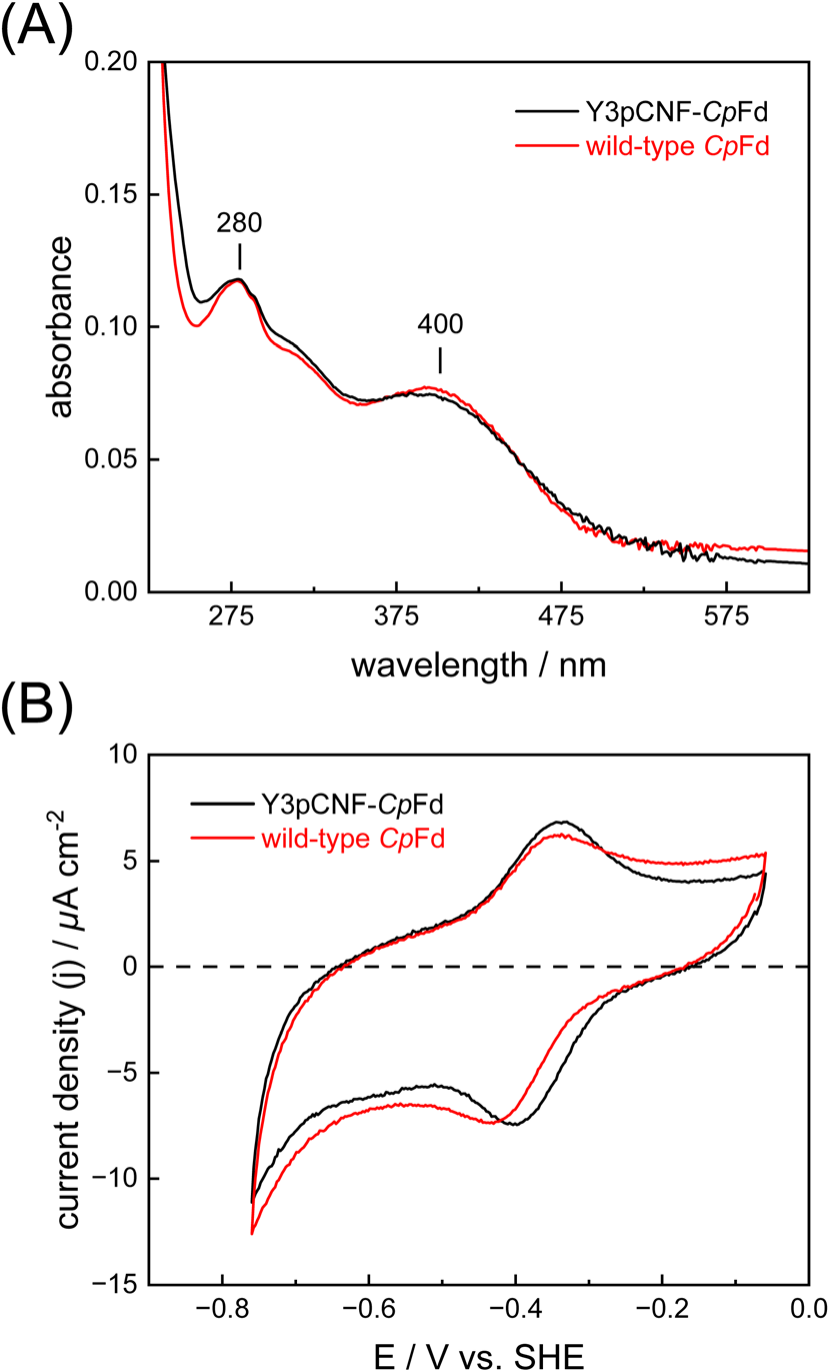
Electronic spectroscopy and protein film electrochemistry. (A) Electronic spectra of wild-type *Cp*Fd (red) and Y3pCNF-*Cp*Fd (black) recorded in aerobic MOPS/NaOH buffer (0.1 M, pH 7) at 25 °C. The broad band at 400 nm shows the presence of oxidized iron–sulfur clusters ([4Fe–4S]^2+^). (B) Representative cyclic voltammograms of wild-type *Cp*Fd (red) and Y3pCNF-*Cp*Fd (black) recorded in aerobic potassium phosphate/NaOH buffer (25 mM pH 7.5, 0.1 M NaCl, and 50 mM MgCl_2_) at 25 °C with a scan rate of 25 mV s^−1^.

The production of holo-*Cp*Fd (wild type) and holo-Y3pCNF-*Cp*Fd in *E. coli* C321.ΔA.opt was confirmed by electronic spectroscopy, where one broad band at ∼400 nm reflects the presence of the oxidized [4Fe–4S] clusters (Fig. 2A).^46^ In comparison to wild-type *Cp*Fd, the total protein yield of Y3pCNF-*Cp*Fd decreased five-fold. This can be explained by translation stalling until the arrival of the pCNF-charged tRNA, or by a low cytoplasmic concentration of free pCNF. Importantly, the production of Y3pCNF-*Cp*Fd was also found to decrease by a further five-fold in the absence of pCNF in the culture medium, confirming the selectivity of the orthogonal aminoacyl-tRNA synthetase/tRNA pair for tRNA charging with pCNF. The incorporation of pCNF was initially verified by fluorescence spectroscopy (Fig. S2†).

We next determined whether the presence of pCNF induces a significant change in *E*^0^′ of its neighboring [4Fe–4S] cluster using protein film voltammetry (Fig. 2B). Due to either (i) fast intermolecular ET between the [4Fe–4S] clusters of *Cp*Fd or (ii) electronically similar environments around the [4Fe–4S] clusters, a single pair of redox peaks at −390 ± 7 mV *vs.* SHE was observed, in good agreement with previous investigations.^47,48^ In the case of the Y3pCNF-*Cp*Fd, a single pair of redox peaks was observed at −378 ± 2 mV *vs.* SHE, suggesting that the presence of pCNF did not introduce a significant change in *E*_m_ (Student's *t*-test, *P* = 0.0996).

**Fig. 3:**
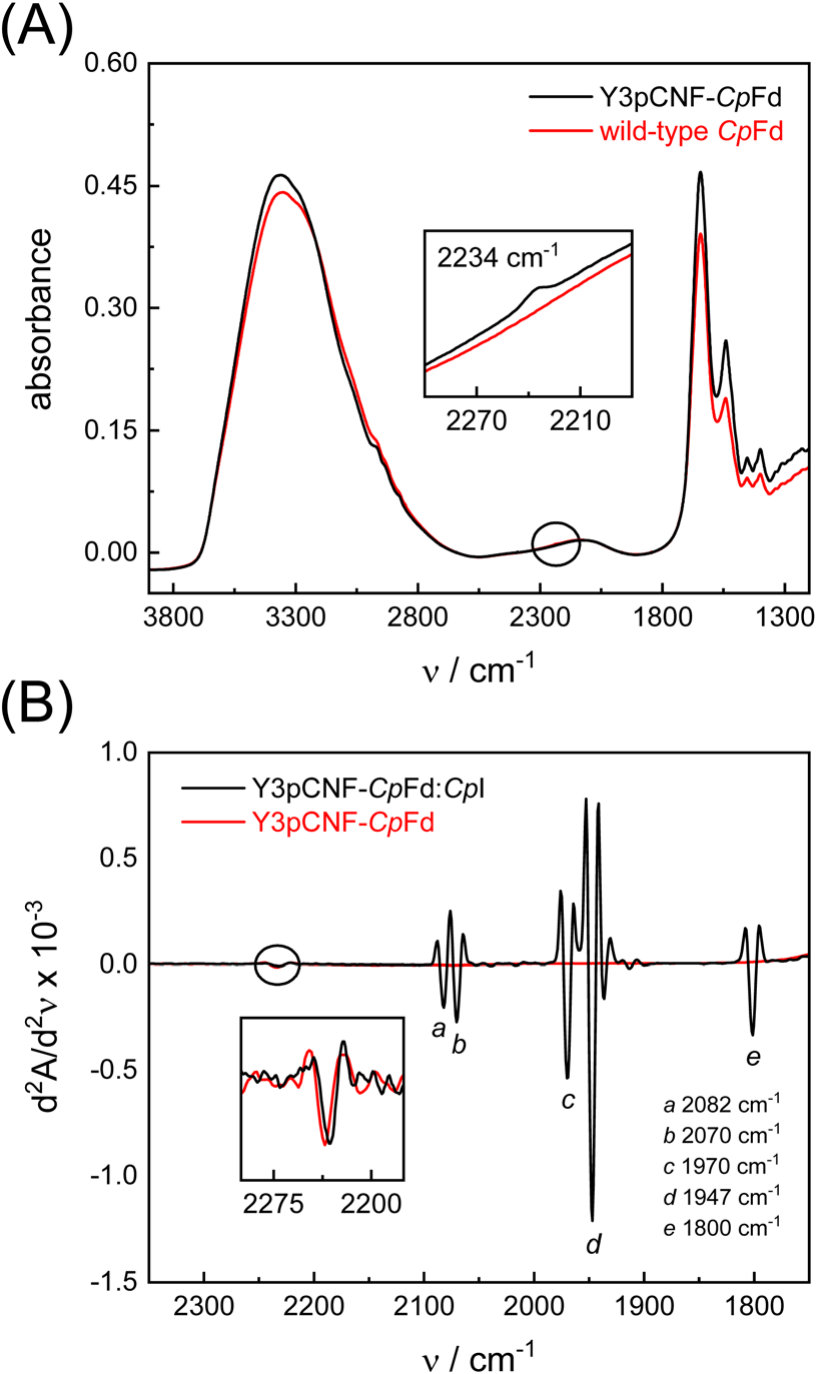
Steady-state vibrational spectroscopy. (A) ATR FTIR spectra of wild-type *Cp*Fd (red) and Y3pCNF-*Cp*Fd (black) confirming the presence of the nitrile band of pCNF at ∼2234 cm^−1^ (inset). (B) Second derivate FTIR spectra of the Y3pCNF-*Cp*Fd:*Cp*I complex (black) show five strong bands (a)–(e) between 2150 and 1750 cm^−1^ that have been assigned to the diatomic ligands of the H-cluster in the oxidized resting state *H*_ox_. These bands are missing in the Y3pCNF-*Cp*Fd spectrum (red); however, at higher frequencies both spectra show the nitrile band of pCNF (inset). In complex with *Cp*I, this band is red-shifted by 3–4 cm^−1^ relative to *Cp*Fd.

The incorporation of pCNF was then verified by two independent vibrational spectroscopic approaches. Raman spectra reveal a single band at 2234 cm^−1^ that is exclusively observed in the Y3pCNF-*Cp*Fd sample and can be assigned to the nitrile stretching vibration of pCNF (Fig. S2†). The relatively high frequency and broad band shape suggests hydrogen-bonding with the solvent.^45^ Independent confirmation was achieved by FTIR spectroscopy (Fig. 3A). Interestingly, we found that the pCNF band position shifted by 4–5 cm^−1^ toward lower frequencies when water was removed from the protein film (Fig. S3†). Therefore, any comparison must be performed under unchanging hydration levels.

No meaningful nitrile shifts were detected upon electrochemical reduction or oxidation, in the presence of the chemical reductant dithionite, or by the oxidation of Y3pCNF-*Cp*Fd with O_2_ (Fig. S4†). Both of these approaches have been exploited to reduce or oxidize ferredoxins in the past.^49–51^ To probe the reduction and oxidation of Y3pCNF-*Cp*Fd under turnover conditions, we mixed the protein with its natural redox partner, the [FeFe]-hydrogenase *Cp*I,^52^ in a ratio of 5 : 1 (1 mM *Cp*Fd and 200 μM *Cp*I). The second derivative FTIR spectrum shows that the nitrile band is found at ∼2230 cm^−1^ (Fig. 3B), suggesting less solvent access around Y3pCNF in the presence of *Cp*I. At lower frequencies, the spectrum reveals strong absorbance bands (a)–(e) that have been assigned to the cyanide (CN^−^) and carbon monoxide (CO) ligands of *Cp*I.^24–26^ The IR signature is characteristic for the oxidized resting state of the H-cluster, *H*_ox_, and unaffected by the presence of *Cp*Fd.

**Fig. 4:**
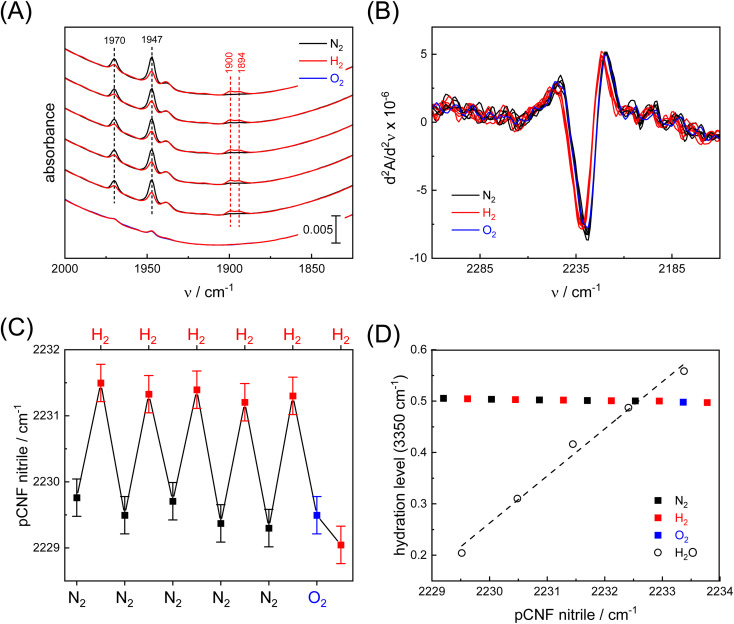
*In situ* analysis of the ferredoxin:hydrogenase complex. (A) ATR FTIR spectra of the H-cluster's CO ligands show the repeated reduction under 10% H_2_ (increase of *H*_red_ and *H*_sred_ at 1900 and 1894 cm^−1^, respectively) and oxidation under N_2_ (*H*_ox_ at 1970 and 1947 cm^−1^). In the presence of 10% O_2_, all CO bands disappear due to disintegration of the H-cluster. (B) Second derivative ATR FTIR spectra of the Y3pCNF-*Cp*Fd:*Cp*I complex in the frequency regime of the nitrile band under N_2_ (black), 10% H_2_ (red), or 10% O_2_ (blue). (C) The shift of the nitrile band as a function of gas composition. After deactivation of *Cp*I with O_2_, a reaction with H_2_ is no longer observed. (D) Shift of the nitrile band as a function of the hydration level (measured at 3350 cm^−1^). The pCNF up-shift for increasingly humid protein films can be followed by linear regression (dashed line, data from Fig. S3†). In comparison, any changes in hydration level during the N_2_/H_2_/O_2_ titration are insignificant, which allows excluding any unspecific pCNF band shifts.

We hypothesize that the reduction and oxidation of *Cp*I would result in ET with Y3pCNF-*Cp*Fd.^30–32^ Therefore, a Y3pCNF-*Cp*Fd:*Cp*I protein film was probed in the presence of N_2_, H_2_, or O_2_ employing our unique setup for *in situ* ATR FTIR spectroscopy.^39^ For this, 1 μL protein solution (200 μM) was concentrated on the ATR crystal under a stream of dry N_2_ and re-hydrated with N_2_ aerosol until the protein film adopted an equilibrium with the humidity in the gas phase. Five N_2_/H_2_ cycles followed by incubation with O_2_ were performed, where a treatment with O_2_ at the end of the experiment deactivates *Cp*I – [FeFe]-hydrogenases are O_2_-sensitive^53^ – ultimately impeding H_2_ oxidation and subsequent ET toward Y3pCNF-*Cp*Fd. To ensure that the system adopted equilibrium conditions, each gas treatment was performed for 180 s before the spectra were compared. The apparent rate constants for reduction and auto-oxidation of *Cp*I are *k*_1_ = 2.8 ± 0.1 s and *k*_–1_ = 51 ± 1.6 s, respectively (Fig. S8†). The data in Fig. 4A illustrate how *H*_ox_ converts into redox states *H*_red_ and *H*_sred_ under 10% H_2_, auto-oxidation in the absence of H_2_ (100% N_2_), and oxygenic deactivation of *Cp*I in the presence of 10% O_2_. Fig. 4B shows the respective second derivative spectra in the energy regime of the pCNF nitrile band (same data set as in panel A). We observe a distinct up-shift from 2229.5 ± 0.15 cm^−1^ to 2231.3 ± 0.15 cm^−1^ in the presence of 10% H_2_ (Δ*ν* = 1.8 cm^−1^) that is reversible when the gas atmosphere is switched back N_2_. Plotting the band position against gas treatment (Fig. 4C) reveals the reversibility of the shift and illustrates that once *Cp*I is deactivated with 10% O_2_, subsequent treatment with H_2_ no longer impacts the nitrile band. Significant hydration changes that would affect the nitrile frequency were avoided (Fig. 4D and S3†).

With a Stark tuning rate of 0.71 cm^−1^ mV^−1^ cm^−1^ for pCNF,^54^ a 1.8 cm^−1^ shift is equivalent to a local electric field change of +2.53 mV cm^−1^, well in agreement with reports for other proteins.^55–57^ The observed up-shift indicates an accumulation of negative charge in front of the nitrile group's N-atom upon reduction, *e.g.*, due to protein structural changes, the reduction of [4Fe–4S] cluster F of *Cp*Fd, or a reduction of [4Fe–4S] cluster C of *Cp*I (Fig. 1). The later interpretation is particularly interesting as Artz *et al.* have argued that cluster C controls hydrogenase activity due to its low *E*^0^′ of approximately −450 mV *vs.* SHE, functioning as a “gate keeper” for catalytic ET.^30^ Whether the nitrile shift is a general feature of hydrogenase reduction or specific to the complex with *Cp*I can be probed in presence of *Cr*HydA1 as an alternative [FeFe]-hydrogenase. The so-called minimal [FeFe]-hydrogenase *Cr*HydA1 lacks the iron–sulfur cluster containing F-domain of *Cp*I (Fig. 1) but has been demonstrated to interact with both plant-type and bacterial ferredoxins.^58–60^ Surprisingly, however, a nitrile shift was not observed when a Y3pCNF-*Cp*Fd:*Cr*HydA1 film was reacted with H_2_ (Fig. S5†). As this argues against a detection of reduced cofactors, we speculate that the Stark shift of the pCNF nitrile band is due to protein structural changes in the Y3pCNF-*Cp*Fd:*Cp*I complex and PPI-coupled ET events specific for the interaction with the F-domain of *Cp*I.

**Fig. 5:**
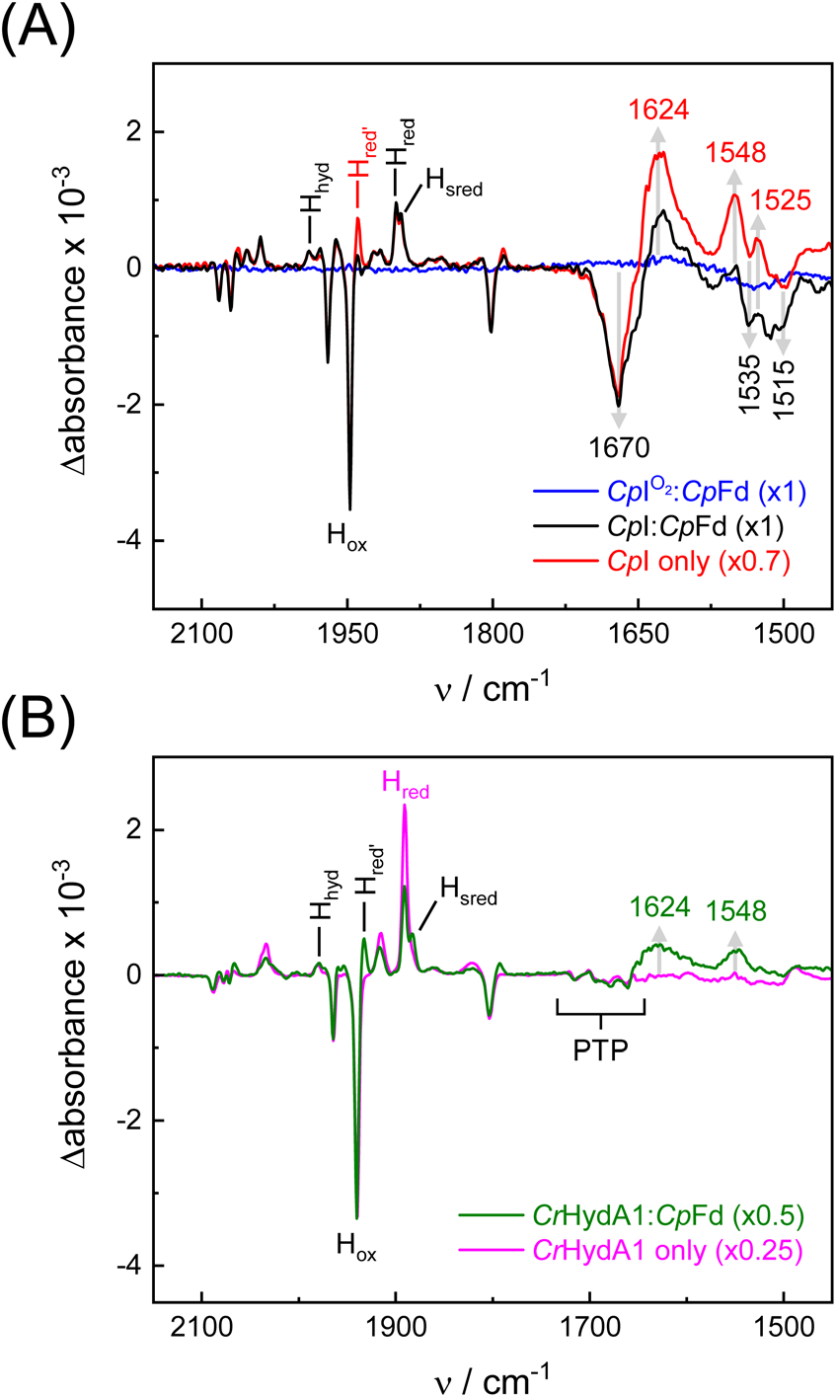
Infrared difference spectroscopy. (A) H_2_–N_2_ ATR FTIR difference spectra of Y3pCNF-*Cp*Fd:*Cp*I (black) and *Cp*I alone (red) after 25 s. Bands above 1750 cm^−1^ are assigned to H-cluster ligands in the *H*_ox_ state (negative) and various reduced states (positive). At lower frequencies, the 1670/1624 cm^−1^ down-shift in the amide I regime indicates protein structural changes, alongside up-shifts at 1535/1548 cm^−1^ and 1515/1525 cm^−1^. The blue spectrum serves as a negative reference where *Cp*I was inactivated with O_2_ ahead of the reaction with H_2_. (B) H_2_–N_2_ ATR FTIR difference spectra of Y3pCNF-*Cp*Fd:*Cr*HydA1 (green) and *Cr*HydA1 alone (magenta) after 50 s. Note the lack of the difference features in *Cr*HydA1 in the amide I regime while only small changes are observed in the presence of Y3pCNF-*Cp*Fd. “PTP” refers to small difference features explored earlier.^62^ All spectra are normalized to the main CO band of *H*_ox_ (1947 cm^−1^ in *Cp*I, 1940 cm^−1^ in *Cr*HydA1) with factors as indicated in the legends.

To investigate redox-dependent protein structural changes, FTIR difference spectra of Y3pCNF-*Cp*Fd in the presence of *Cp*I or *Cr*HydA1 were analyzed. Spectra under N_2_ were subtracted from spectra under 10% H_2_ yielding “H_2_–N_2_” difference spectra recorded in a time-dependent fashion. Fig. S6† shows the temporal evolution of H_2_–N_2_ difference spectra for Y3pCNF-*Cp*Fd:*Cp*I, 5–25 s after initial contact with H_2_. These data depict the conversion of steady-state oxidized (equilibrium under N_2_) into steady-state reduced (equilibrium under H_2_) and as such are unrelated to the microscopic velocity of complex formation or catalysis. We emphasize that transient modulations of the ferredoxin:hydrogenase complex cannot be visualized by this approach. Fig. 5A shows H_2_–N_2_ difference spectra after 25 s for different samples. Between 2150 and 1750 cm^−1^, negative bands indicate depletion of the oxidized H-cluster (*H*_ox_, as accumulated under N_2_) while positive bands indicate evolution of the reduced H-cluster states *H*_red_ and *H*_red′_ as well as *H*_sred_ and *H*_hyd_ in the presence of H_2_ (Table S2†).^61^ Below 1700 cm^−1^, the spectra show differential signals at 1670 and 1624 cm^−1^ as well as 1548 and 1535 cm^−1^. A negative band pattern becomes visible around 1515 cm^−1^. The prominent 1670/1624 feature can be assigned to secondary structural changes involving the amide I band, *i.e.*, a formation of β-sheets over random coil structures.^34^ Importantly, as the amide I band is dominated by the C=O stretching frequency of the protein backbone, the lack of spectral shifts in D_2_O is in agreement with this assignment (Fig. S6†). Moreover, the unchanging band position at 1670 cm^−1^ in D_2_O allows a decrease in the hydration level to be excluded (Fig. S6† additionally shows how spectral differences in the OH stretching regime of water were negligible). Smaller features below 1600 cm^−1^ might stem from amide II changes; however, no significant downshifts were observed upon deuteration. A reference experiment with Y3pCNF-*Cp*Fd and O_2_-deactivated *Cp*I demonstrates that the formation of any difference feature below 1700 cm^−1^ is dependent on hydrogenase activity; the blue trace in Fig. 5A was calculated from the final H_2_ treatment in Fig. 4C and basically resembles a baseline.

Further exploring the origin of protein structural changes, we probed *Cp*I in the absence of ferredoxin (Fig. 5A). Surprisingly, a very similar difference spectrum was observed, with slightly more pronounced difference features at 1548/1535 cm^−1^ and 1525/1515 cm^−1^ as well as a greater accumulation of the *H*_red′_ state, indicative of a fully reduced composition of [4Fe–4S] clusters.^63^ This comparison permits the observed changes to be assigned to the hydrogenase rather than the ferredoxin. A similar interpretation was proposed by Voloshyn *et al.*, detecting structural changes in the “sensory” [FeFe]-hydrogenase from *Thermoanaerobacter mathranii* upon reduction.^64^ As the Stark shift of Y3pCNF-*Cp*Fd is observed exclusively in complex with *Cp*I (Fig. S4 and S5†), we speculate that the nitrile group near the [4Fe–4S] cluster of ferredoxin reports on changes in protein structure rather than redox state. Therefore, we analyzed the H_2_–N_2_ difference spectrum of a Y3pCNF-*Cp*Fd:*Cr*HydA1 protein film in the next step. Band changes below 1700 cm^−1^ were nearly absent (the green traces in Fig. 5B only show small positive bands), and not detected at all when *Cr*HydA1 was reduced in the absence of *Cp*Fd. These findings permit the origin of protein structural changes to be localized: assuming that the iron–sulfur clusters of the F-domain in *Cp*I mediate ET between H-cluster and ferredoxin^30–32^ while the H-cluster in *Cr*HydA1 interacts with ferredoxin directly,^65–67^ our data now strongly suggest that protein structural changes of the F-domain modulate PPIs and ET between hydrogenase and ferredoxin.

**Fig. 6:**
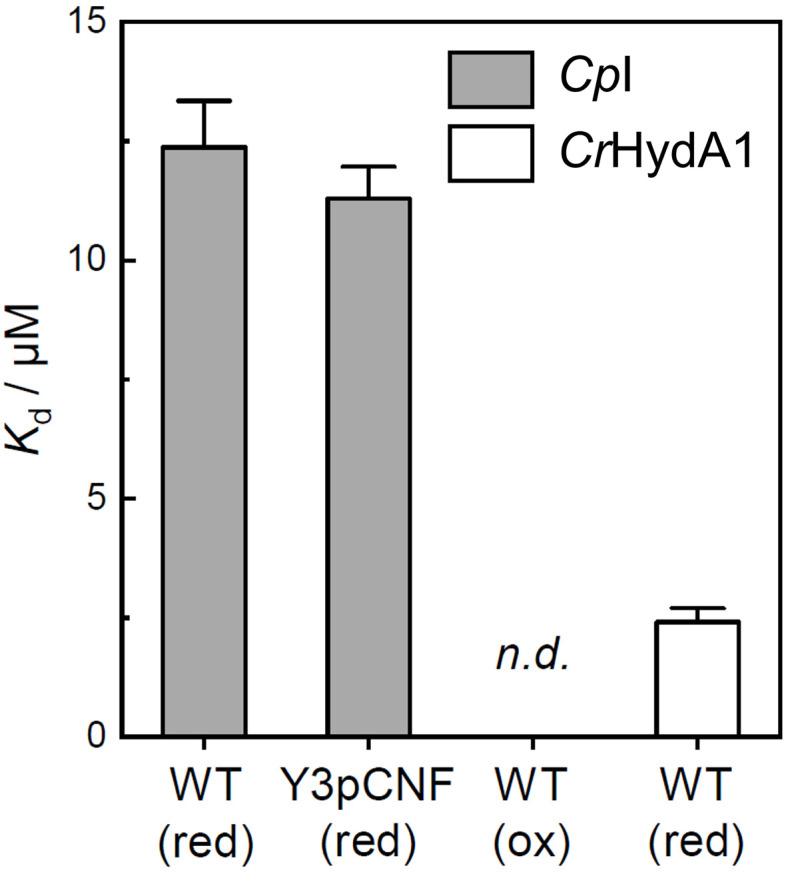
Microscale thermophoresis. Dissociation constants (*K*_d_) of the *Cp*Fd:*Cp*I (WT) and Y3pCNF-*Cp*Fd:*Cp*I complex under reducing or oxidizing conditions (red/ox). Under oxidizing conditions in the absence of dithionite, an interaction between *Cp*Fd:*Cp*I was not detected (n.d.). The open bar depicts the *K*_d_ of the *Cp*Fd:*Cr*HydA1 complex under reducing conditions. See Fig. S7† for the titration curves and additional data.

Whether reduction leads to a formation or dissociation of the ferredoxin:hydrogenase complex is unclear from the FTIR data. Therefore, we performed anaerobic microscale thermophoresis (MST) analyses to compare the dissociation constants (*K*_d_) of *Cp*I with *Cp*Fd or Y3pCNF-*Cp*Fd under reducing or oxidizing conditions. In the presence of hydrogenase, the characteristic dithionite-bleach^68^ of the Alexa Fluor 647 dye used for labeling ferredoxin was found to be reversible and facilitated a comparison of *K*_d_ for *Cp*Fd:*Cp*I and Y3pCNF-*Cp*Fd:*Cp*I under reducing conditions (Fig. 6 and S7†). This “protection” of the Alexa Fluor 647 label on ferredoxin was specific to *Cp*I and not observed with other proteins (Fig. S7†). From a fit of the data, a *K*_d_ of 12.4 ± 0.97 μM was derived for wild-type *Cp*Fd and a similar *K*_d_ of 11.3 ± 0.71 μM was obtained for Y3pCNF-*Cp*Fd, suggesting that the mutation does not alter the interaction with *Cp*I. These values are in excellent agreement with *K*_d_ values obtained for solution-based H_2_ evolution assays of *Cp*I and dithionite-reduced *Cp*Fd (16.5 ± 5 μM).^69^ Quinkal and co-workers reported a maximal H_2_ evolution rate (*V*_max_) of 720 μmol H_2_ per min per mg, which underscores the kinetic relevance of the *K*_d_ values determined by MST here. Notably, no significant interaction could be detected between oxidized wild-type *Cp*Fd and *Cp*I (Fig. S7†), cementing the importance of redox states in PPIs. The clostridial ferredoxin has been shown to support proton reduction with other [FeFe]-hydrogenases, *e.g.*, *Cr*HydA1.^70^ The low *K*_d_ of 2.41 ± 0.29 μM obtained from our MST experiments (Fig. S7†) supports this observation and justifies comparing *Cp*Fd:*Cp*I and *Cp*Fd:*Cr*HydA1 in the FTIR experiments. Note that *Cr*HydA1 forms an ever stronger complex with its natural redox partner, PetF (Fig. S7†).

Oxidizing conditions in the MST experiment resemble the equilibrium under N_2_ in the FTIR experiment as most [FeFe]-hydrogenases convert into the oxidized state *H*_ox_ in the absence of external reductants.^71^ Equally, reducing conditions in the MST experiment resemble the equilibrium under H_2_ in the FTIR experiment. Both dithionite and H_2_ are commonly used as reductants for hydrogenases, although H_2_ may be the preferable agent.^72,73^ Based on these conservative assumptions, the H_2_–N_2_ difference spectra in Fig. 5A can now be explained with complex formation upon reduction of *Cp*I and *Cp*Fd in the presence of H_2_. The negative bands at 1670, 1535, and 1515 cm^−1^ are assigned to the F-domain in the “free”, oxidized form while positive bands at 1624, 1548, and 1525 cm^−1^ are assigned to the F-domain in the ferredoxin-bound, reduced form. As emphasized above, our data do not directly reflect the transient modulation of the ferredoxin:hydrogenase complex; however, an underlying mechanism presents itself. We speculate that redox-dependent protein structural changes facilitate complex formation and intermolecular ET in the presence of reducing equivalents. Once oxidized – *e.g.*, upon H_2_ evolution – the complex reverts into the oxidized structure and releases the ferredoxin. The distal [4Fe–4S] cluster of the F-domain (C in Fig. 1) must play a crucial role as level indicator in this mechanism, as emphasized by Artz *et al.* earlier.^30^ Managing ET *via* the F-domain may explain the superior catalytic activity of bacterial [FeFe]-hydrogenases like *Cp*I over algal [FeFe]-hydrogenases like *Cr*HydA1.

## Conclusions

In summary, we present a dynamic analysis of the electron transfer complex between ferredoxin *Cp*Fd and the structurally diverse [FeFe]-hydrogenases *Cp*I and *Cr*HydA1. The introduction of the non-canonical amino acid pCNF as a Stark probe permitted electric field changes upon reduction or oxidation of the complex to be determined, and FTIR difference spectroscopy uncovered pronounced protein structural changes within the F-domain of *Cp*I. Such dynamic behavior is rarely picked up in structural biology, although the “sampling” of different conformations in cryogenic electron microscopy (cryo EM) may lead to respective findings.^74–76^ The MST affinity analysis of *Cp*Fd and *Cp*I confirmed that the observed electric field and protein structural changes reflect a formation of the protein–protein complex under reducing conditions, prompted by redox-dependent PPIs. We speculate that the *Cp*Fd:*Cp*I complex may be favored when redox states are complementary, *i.e.*, oxidized *Cp*I may “select” for reduced *Cp*Fd, and *vice versa* (most [FeFe]-hydrogenases are bidirectional^77^). Similar concepts of interprotein ET have been discussed early on.^78^

Our observations now explain the typically superior performance of hydrogenases with ferredoxin over general reductants. On the one hand, the specificity of contact secures optimal ET distances between the iron–sulfur clusters, as sensed by the local Stark probe pCNF. On the other hand, global changes at the ET interface allow dissociation of the complex once electron transfer has taken place, *i.e.*, upon oxidation. This can lead to an oscillation between “on and off” when the ferredoxin pool, an important metabolic marker,^79–81^ is sufficiently reduced. This way, the two-electron chemistry of hydrogenase is promoted by a one-electron redox partner like ferredoxin.

We note that two-electron redox chemistry with 2[4Fe–4S]-type ferredoxins (*i.e.*, proceeding *via* two one-electron transfers) cannot be strictly excluded. A single interaction between *Cp*I and *Cp*Fd may function to transfer a total of two electrons, given that the two [4Fe–4S] clusters are of similar potential and situated within physiologically relevant ET distance.^82^ However, evidence for redox-dependent PPIs in the interaction with ferredoxin can be found in a wide range of systems, hinting at a general phenomenon. For example, the [2Fe–2S]-type ferredoxin of *Anabaena* PCC7119 undergoes distinct changes upon reduction,^83^ and when Kurisu *et al.* crystallized the first ferredoxin-dependent protein complex in 2001,^84^ the authors highlighted structural changes in both ferredoxin and Fd:NADP^+^ reductase (FNR). The importance of such changes was rationalized by Xu *et al.* who used paramagnetic NMR spectroscopy to investigate the ternary complex between ferredoxin and Fd:Trx reductase (FTR) that “catalyzes” reduction of thioredoxin (Trx) eventually.^85^ Recent data from Steinhilper *et al.* suggests that similar principles may apply to other ferredoxin-driven redox processes as well.^86^

## Data availability

The data supporting this article have been included as part of the ESI.†

## Author contributions

The manuscript was written through contributions of all authors. All authors have given approval to the final version of the manuscript.

## Conflicts of interest

The authors declare no conflict of interest.

## Supplementary Material

SC-OLF-D5SC00550G-s001

SC-OLF-D5SC00550G-s002
