# Supplementary material for: Probing the ferredoxin:hydrogenase electron transfer complex by infrared difference spectroscopy
Source: Chem Sci. 2025 Apr 30;16(23):10465–75. doi: 10.1039/d5sc00550g (PMC12067460; doi:10.1039/d5sc00550g)
Supplement: SC-016-D5SC00550G-s002 [file SC-016-D5SC00550G-s002.pdf]

Supplementary Information (SI) for Chemical Science.
This journal is © The Royal Society of Chemistry 2025

Supporting Information for

Probing the Ferredoxin:Hydrogenase Electron Transfer Complex by Infrared Difference Spectroscopy

Selmihan Sahin, Johanna Brazard, Takuji B. M. Adachi, Ulrich Mühlenhoff, Ross D. Milton*, Sven T. Stripp*

CONTENTS

- **Material and Methods**
- **Figure S1.** Close-up of the computed *Cp*Fd:*Cp*I interface.
- **Figure S2**. Fluorescence and Raman spectroscopy.
- **Figure S3.** Shift of the nitrile band as a function of hydration level.
- **Figure S4.** Reduction and oxidation of ferredoxin.
- **Figure S5.** Shift of the nitrile band as a function of redox conditions.
- **Figure S6.** Further evaluation of secondary structural changes.
- **Figure S7.** MicroScale Thermophoresis.
- **Figure S8.** Time frame of reduction and auto-oxidation
- **Table S1.** Calculated yields of *Cp*Fd and Y3pCNF-*Cp*Fd protein production, including A_{390}/A_{280} ratios.
- **Table S2.** H-cluster bands of *Cp*I and *Cr*HydA1 in the H_{ox} and H_{red} states.
- Supplementary Bibliography

MATERIALS AND METHODS

Strains, plasmids, and reagents. Amber codon suppression was performed using *Escherichia coli* strain C321.ΔA.opt, in which all amber codons have been re-coded (strain C321.ΔA.opt was a gift from George Church, Addgene plasmid #87359).(1) Site-specific pCNF insertion by amber codon suppression was performed using the aminoacyl tRNA synthetase/tRNA pair offered by the plasmid pDule2-pCNF, a gift from Ryan Mehl (Addgene plasmid #85495).(2) Initial pCNF incorporation tests were performed using plasmid pBAD-sfGFP 150TAG, where induction using arabinose yields superfolder green fluorescent protein ("sfGFP") containing the pCNF residue at position 150. This plasmid was linearized by PCR and a synthetic gene encoding wild-type *Cp*Fd carrying a *C*-terminal Strep-tag "WSHPQFEK" (codon optimized, ThermoFisher Scientific Switzerland) was inserted by Gibson assembly to yield plasmid pBAD-*Cp*Fd. pBAD-sfGFP 150TAG was a gift from Ryan Mehl (Addgene plasmid #85483).(3) Note that the Step-tag includes a *C*-terminal tryptophan residue (W) which is not expected to impact the *Cp*Fd:*Cp*I complex (**Fig. S1**). Site-directed mutagenesis was performed using Gibson assembly to replace the codon corresponding to Y3 of *Cp*Fd (TAT) with the TAG amber stop codon, yielding plasmid pBAD-*Cp*Fd-Y3pCNF. The unnatural amino acid pCNF was purchased from Bachem (Switzerland, product 4028063, H-4-cyano-Phe-OH). Unless stated otherwise, all chemicals were purchased from Sigma Aldrich, Inc. (Switzerland) and used without further purification. PCR was performed with Phusion High Fidelity DNA polymerase (Thermo Fisher Scientific Switzerland). Site directed mutagenesis was performed with GeneArt Gibson Assembly HiFi Master Mix (Thermo Fisher Scientific Switzerland) using the specific Gibson primers. Plasmid DNA isolation was performed with the Nucleobond Xtra Mini Kit (Macherey-Nagel, Switzerland). Oligonucleotides were supplied by Microsynth (Switzerland).

Cell growth and protein Purification. *E. coli* C321.ΔA.Opt was chemically transformed with the plasmid pBAD-*Cp*Fd for the production of wild-type *Cp*Fd. To produce pCNF-containing *Cp*Fd, plasmid pBAD-*Cp*Fd-Y3pCNF was chemically co-transformed (sequentially) with plasmid pDule2-pCNF into *E. coli* C321.ΔA.opt cells; successful transcription, translation, and TAG-suppression yields Y3pCNF-*Cp*Fd, where the Tyr3 amino acid has been replaced with pCNF. Glycerol stocks of transformed cells were prepared and kept at −80 °C for further studies.

Starter cultures of the transformed cells were individually prepared in 60 mL of LB-Miller medium supplemented with ferric ammonium citrate (2 mM) and MOPS/NaOH buffer (100 mM, final pH

of 7.8).(4) Starter cultures were incubated at 30 °C (200 rpm) for 18 h of growth. All cultures/media were supplemented with the relevant antibiotics for plasmid selection: 100 μg/mL ampicillin (for pBAD-X plasmids) and 100 μg/mL spectinomycin for pDule2-pCNF. The starter cultures (60 mL) were used to inoculate 3 L of medium in 5 L baffled flasks. Once the optical density had reached $0.5_{600\ nm}$, expression was induced with the addition of arabinose (final concentration = 0.1 % w/v), cysteine was added (to 2 mM), the temperature was lowered to 25 °C, and incubation under microaerobic conditions was achieved by lowering the rpm to 75, overnight. For the expression of Y3pCNF-*Cp*Fd, pCNF was included in the main culture (1 mM) at the time of induction. All cells were harvested by centrifugation at 4000 x *g* for 20 minutes (ambient temperature).

All purification steps were performed under an anoxic environment (within an anoxic glovebox, >95% N_2/<5% H_2, COY Laboratory Products MI USA). All solutions were equilibrated with the glovebox atmosphere by stirred for 24 h. For protein purification, the obtained cell paste was resuspended in "Buffer A" (50 mM potassium phosphate/NaOH buffer, pH 7.0, containing 150 mM NaCl and 0.5 mM DT) containing ~2 μg/mL DNAse A and 1 mg/mL of lysozyme. The resuspended cells were lysed anaerobically by sonication (10 min, 2 sec ON, 2 sec OFF, 50% Amplitude, Fisherbrand FB120) and the cell lysate was clarified by centrifugation at 30,000 x *g* for 1 h at 4 °C to remove cell debris. The supernatant was then filtered through a 0.45 μm syringe filter, and loaded onto a Strep column (StrepTrap XT, 5 mL, Cytiva), pre-equilibrated with 50 mM phosphate/NaOH buffer (pH 7.0 containing 150 mM NaCl and 0.5 mM DT). The column was then washed with 3 column volumes of Buffer A. The wild-type *Cp*Fd or Y3pCNF-*Cp*Fd was subsequently eluted using Buffer A containing 50 mM biotin (Chemodex). The eluted protein was next desalted using a HiPrep 26/10 desalting column pre-equilibrated with Buffer A to remove excess biotin. The eluted proteins were concentrated to 1 mM using an Amicon stirred concentrator cell (Merck-Millipore) equipped with a 3 kDa microfilter. The concentrated protein was then stored as 10 μL pellets in liquid nitrogen until further use. Protein concentrations of the preparations were estimated using a molar absorptivity of $\varepsilon_{390} = 30\ mM^{-1}\ cm^{-1}$ and relative iron-sulfur cluster content was estimated considering the ratio $A_{390\ nm}/A_{280\ nm}$ for *Cp*Fd which had oxidized in air.(5) The obtained data suggest excellent purity. We acknowledge Thomas Happe for providing a sample of ferredoxin PetF from *C. reinhardtii*.(6)

[FeFe]-hydrogenases *Cp*I and *Cr*HydA1 were produced as reported previously.(4) In brief, hydrogenase genes carrying a C-terminal Strep-tag were heterologously produced in *E. coli* BL21

ΔiscR carrying plasmid pACYC-hydEF-hydGX for the co-expression of [FeFe]-hydrogenase-specific maturases HydEFG.(7) Following strep-tag affinity purification, samples were concentrated to 200 µM and stored anaerobically. *Cp*I and *Cr*HydA1 sample purity was documented in earlier work.(8, 9)

In silico Structure Prediction. The tertiary structure of *Cp*Fd containing a C-terminal StrepTag (WSHPQFEK) was predicted by AlphaFold using the ChimeraX plugin.(10) The distance between tyrosine Y3 and tryptophan W57 (*i.e.*, between O and N of the side chains) was calculated using ChimeraX. The model is in good spatial agreement with the NMR structure of *Cp*Fd (PDB ID 1CLF). The protein-protein docking model for *Cp*I (PDB ID 6N59) and Strep-tagged *Cp*Fd was then generated by ClusPro.(11) The structure of *Cp*I was fitted over the model to reintroduce the cofactors (identical fit). The NMR structure of *Cp*Fd was overlaid using the MatchMaker function in ChimeraX. For the *Cr*HydA1:PetF complex, we used the computed coordinates of the *Cr*HydA2:PetF complex published by Chang *et al.* in 2007 (12). A SWISS-MODEL (13) homology structure for *Cr*HydA1 was used to replace *Cr*HydA2, and the NMR structure for PetF (PDB ID 2MH7) was used to replace the homology model of PetF.

Fluorescence Measurements. Fluorescence emission spectra were recorded on a TECAN infinite M Nano+ absorbance plate reader using a black plate with an integration time of 20 µs nm^{-1}, a resolution of 2 nm, and an excitation slit width of 5 nm. The excitation wavelength of pCNF (50 µM) was determined by scanning the wavelength range of 230–260 nm, where the maximum excitation wavelength was determined as 238 nm. Fluorescence emission spectra were recorded between 280 nm and 450 nm by exciting 10 µM protein samples in 0.1 M MOPS/NaOH buffer (pH 7.0) at 238 nm. For urea-unfolded protein spectra, the protein samples were treated with 8 M urea for 2 h at 37 °C.

Raman spectroscopy. Raman spectra of wild-type *Cp*Fd and Y3pCNF-*Cp*Fd were recorded at room temperature, using a home-built microspectroscopy setup as described previously.(14) The excitation source was a 532 nm CW laser (Laser Quantum, Opus 532). The laser beam was depolarized using a liquid crystal polymer depolarizer (Thorlabs, DPP25-A). The beam was reflected by a dichroic beamsplitter (AHF Analysentechnik AG, Raman beamsplitter RT 532 rdc). The beam was focused at 50 µm from the glass substrate into a sample by the water-immersion objective lens (Olympus UPLSAPO60XW, NA=1.2). The laser power was ~20 mW after the objective lens. The collected signal was spatially filtered at the conjugate plane using a 50 µm

pinhole and a 532 nm RazorEdge ultrasteep long-pass filter (Semrock, LP03-532RE-25) was used to remove the excitation beam from Raman scattering. Raman spectra were acquired by using a spectrograph (Andor, Kymera 328) with an EMCCD (Andor, Newton 970). Each spectrum was recorded by averaging seven spectra with the exposure time of 360 s (total accumulated time of 42 min). The averaged spectra were smoothed by using a Savitzky-Golay filter. 10 μL of the protein in buffer (~1 mM) was deposited on a clean cover glass with a silicone isolator sheet (0.25 mm thick, Grace Bio-Labs 664475) with a 10 mm hole. The samples were sealed by adding a cleaned cover glass on top of the silicone isolator sheet.

Infrared spectroscopy. All experiments on *Cp*Fd and [FeFe]-hydrogenases *Cp*I and *Cr*HydA1 were performed on hydrated protein films in attenuated total reflection (ATR) configuration using a FTIR spectrometer (Bruker Tensor27) equipped with an MCT detector cooled by liquid N_2.(15) All data were recorded with a spectral resolution of 2 cm^{-1} at 80 kHz scanning velocity. For 50 co-additions of interferometer scans in forward/backward direction, a temporal resolution of 10 s was achieved. Steady-state spectra represent co-addition of up to 1.000 interferometer scans.

All experiments were conducted under 1 atm N_2, at ambient temperature, and in the dark. Reduction of *Cp*I was triggered by introducing 10% H_2 in the gas phase while pure N_2 induced catalytic "auto-oxidation" of *Cp*I. In the presence of 10% O_2, the [FeFe]-hydrogenase was deactivated.(16) The redox-dependent pCNF frequency shifts were analyzed comparing second derivative absolute spectra as calculated in OPUS (Bruker). Secondary structural changes in the *Cp*Fd:*Cp*I complex were evaluated by subtracting spectra under N_2 from spectra under 10% H_2 (in the presence of H_2O or D_2O).

Protein film electrochemistry. All electrochemical measurements were performed by using an AUTOLAB PGSTAT101 controlled by NOVA (Metrohm Suisse), connected to an anoxic Ar glove box (< 1 ppm O_2, Jacomex, France). *Cp*Fd and Y3pCNF-*Cp*Fd bioelectrodes were prepared by drop-casting 2 μL of either protein (250 μM stock solutions, approximately 0.2 nmol) on 3 mm diameter graphite rod electrodes (0.07 cm^{-2}, prepared by heat-shrink insulating walls of the electrode). The electrodes were left to dry under Ar at room temperature for 10 min.

All cyclic voltammograms (CVs) were recorded in 25 mM potassium phosphate/NaOH buffer (pH 7.5, containing 0.1 M NaCl and 50 mM $MgCl_2$) using a scan rate of 25 mV/s alongside a platinum wire counter electrode and a saturated calomel electrode (SCE) as the reference electrode. All

potentials were converted to the standard hydrogen electrode (SHE) according to $E_{SHE} = E_{SCE}$ +0.242 V. GNU Octave was used to remove the background applying a sixth-order spline.

Reference experiments showed that the ferredoxin redox response exclusively stems from drop-cast protein. CVs recorded in *Cp*Fd solution between 0.2–2 nmol did not yield any redox peaks. The protein is clearly electrode-surface confined, albeit relatively weakly as washing the electrode surface with buffer is sufficient to remove *Cp*Fd.

MicroScale Thermophoresis. For MicroScale Thermophoresis (MST) *Cp*Fd samples were labeled with a two-fold molar excess of Sulfo-NHS Alexa Fluor 647 dye (Thermo) for 30 min at room temperature in 50 mM phosphate buffer (pH 7.4) with 50 mM NaCl. The reaction was quenched by the addition of 50 mM TRIS buffer (pH 8) and non-incorporated label was removed using Zebra Spin desalting columns (Thermo). Labeled *Cp*Fd proteins (200 nM) were titrated with 1:1 serial dilutions of unlabeled *Cp*I and *Cr*HydA1 protein (from 100 µM to 3 nM) in 50 mM TRIS buffer (pH 8) with 50 mM NaCl, 1 mM $MgCl_2$, 0.05 mg/mL BSA, and 0.05% Tween20. All buffers, samples, and MST accessories were stored and prepared under strictly anaerobic conditions (95% N_2, 5% H_2, <2 ppm O_2) inside a Coy Laboratories glovebox. The O_2 content was continuously monitored. Residual H_2 in the samples without dithionite may lead to partly reduced hydrogenase; however, **Fig. SX** shows that *Cp*I quickly consumes H_2, adopts a mixture of reduced states, and afterwards converts back into the oxidized state within 2–3 minutes. At the moment of the MST experiment, it is reasonable to assume that all samples without dithionite represent *Cp*I and *Cp*Fd in the oxidized state. Thermophoresis assays were performed using the Monolith NT.115 device (NanoTemper) at 22°C (LED power between 20% and 40%, IR laser power 80%) in standard glass capillaries. Due to their low oxygen transmission rate, these glass capillaries can be used outside of the glovebox.(17) At least three independent experiments were recorded. Experimental data were processed by Nano Temper Analysis 1.5 to estimate K_d values.

Supporting Figures

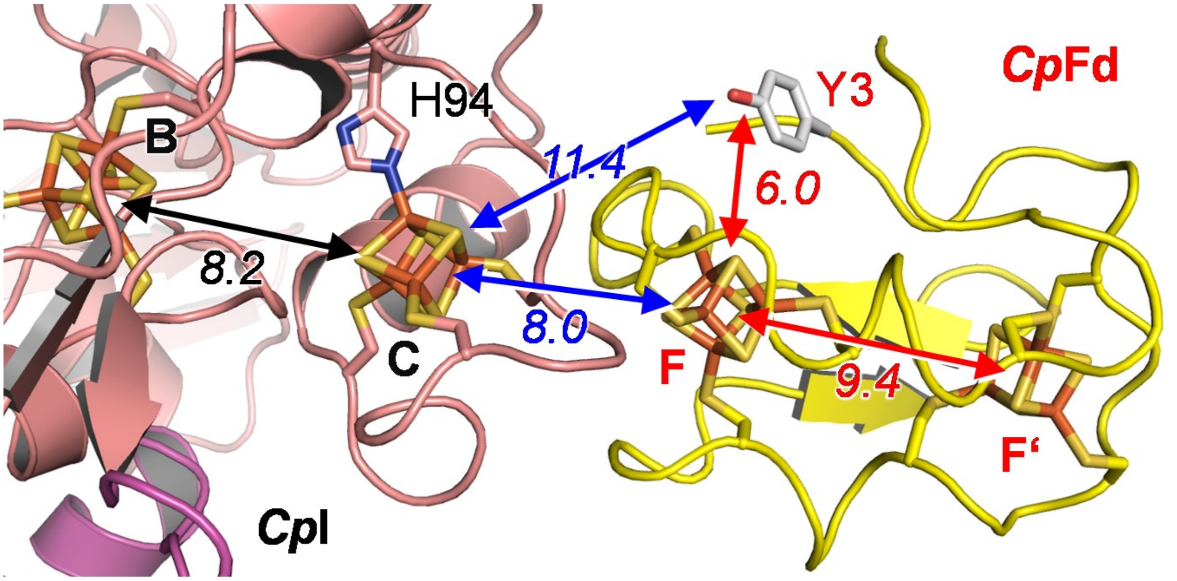

Figure S1. Close-up of the computed *Cp*Fd:*Cp*I interface. The F-domain of [FeFe]-hydrogenase *Cp*I (PDB ID 6N59, including iron-sulfur clusters B and C) is shown in pink cartoon, ferredoxin *Cp*Fd (PDB ID 1CLF, including iron-sulfur clusters F and F´) is shown in yellow cartoon. Further atoms are shown in blue (N), red (O), yellow (S), and orange (Fe) sticks. Tyrosine Y3 is exchanged to cyano-phenylalanine (pCNF, white sticks). All edge-to-edge distances are given in angstrom (*Cp*I intramolecular in black, *Cp*Fd intramolecular in red, *Cp*I:*Cp*Fd intermolecular in blue). Note that [4Fe-4S] cluster C in *Cp*I is naturally coordinated by a loop of one histidine (H94) and three cysteines (C98, C101, C107),

The noncanonical amino acid pCNF is of interest due to improved molar absorptivity and fluorescence quantum yield over tryptophan (W) or tyrosine (Y), serving as an optical probe to characterize conformational changes in proteins.(5) However, if the distance between W or Y and pCNF is sufficiently small, fluorescence quenching through Förster Resonance Energy Transfer (FRET) occurs.(18, 19) In the case of Y3pCNF-*Cp*Fd, the only tyrosine residue of the protein was replaced with pCNF (Y3, see **Fig. S1**) but a *C*-terminal W residue was introduced as part of the Strep-tag. A prediction of the tertiary structure of Y3pCNF-*Cp*Fd by AlphaFold (10) suggests that the distance between pCNF and W could be as little as 7.6 Å; a suitable distance for FRET. **Figure S2** reports the fluorescence emission of the pCNF residue of Y3pCNF-*Cp*Fd, where protein unfolding in the presence of 8 M urea results in an increase of fluorescence emission of pCNF. This is consistent with quenching of pCNF emission when the protein is in a folded state and pCNF is presumably closer to W than in the unfolded state. The increase in fluorescence emission can also be explained by an increase in solvation of the pCNF residue upon protein unfolding.

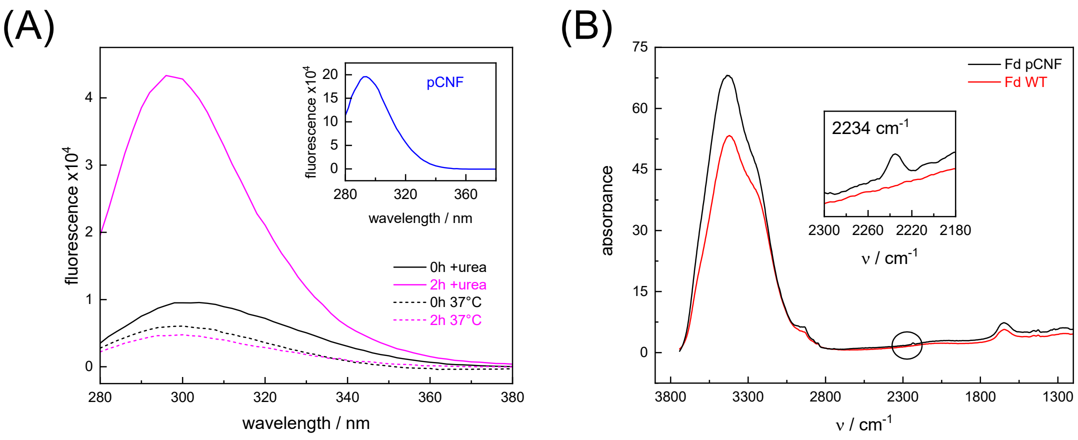

Figure S2. Fluorescence and Raman spectroscopy. (A) Fluorescence spectra of Y3pCNF-*Cp*Fd in the folded (0 h with 8 M urea, black solid line) and unfolded state (2h at 37 °C with 8 M urea, magenta solid line). Dashed lines depict emission spectra of Y3pCNF-*Cp*Fd without urea at an elevated temperature of 37 °C after 0 h and 2 h incubation time (black and magenta line, respectively). Inset: Fluorescence spectrum of pCNF (50 µM) in water. Y3pCNF-*Cp*Fd was prepared in 100 mM MOPS pH 7.0 with a concentration of 10 µM. Fluorescence was monitored between 280–380 nm with an excitation wavelength of 238 nm. **(B)** Raman spectra of ~1 mM wild-type *Cp*Fd (red) and Y3pCNF-*Cp*Fd (black) acquired at room temperature, confirming the presence of the nitrile band of pCNF at ~2234 cm^{-1} (inset).

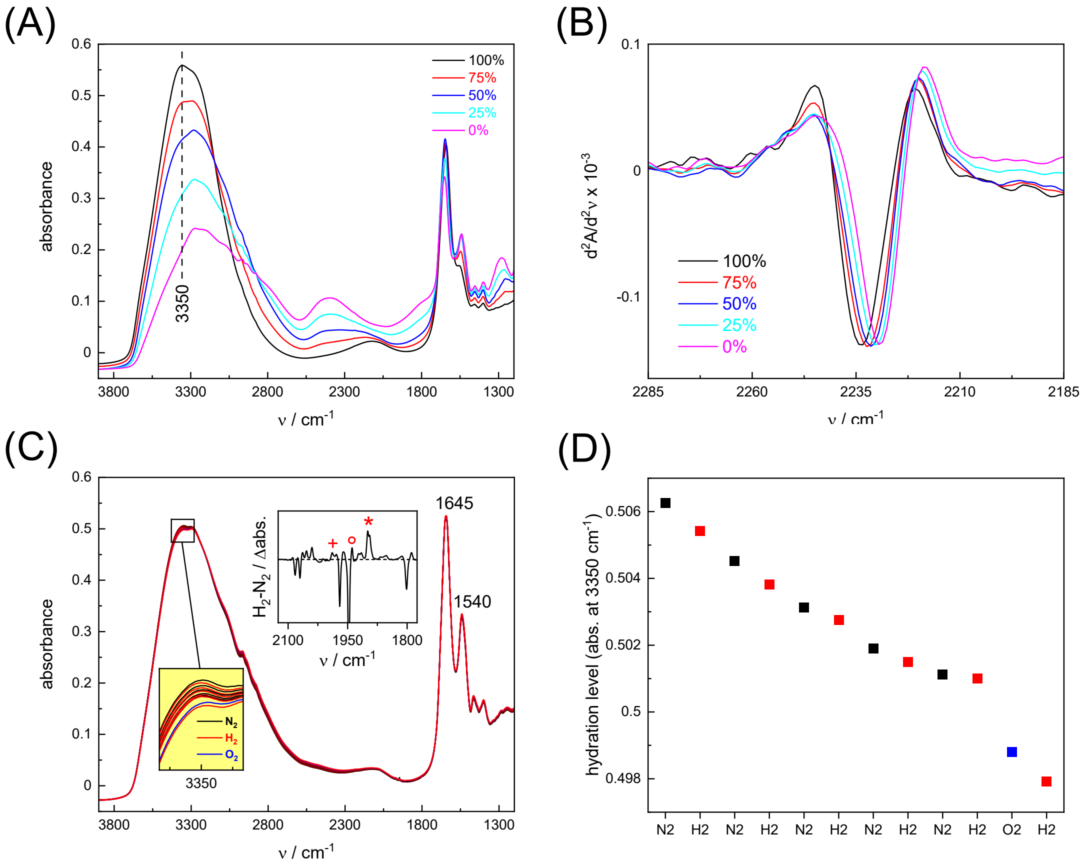

Figure S3. Shift of the nitrile band as a function of hydration level. (A) ATR FTIR spectra of ~1 mM Y3pCNF-*Cp*Fd acquired at room temperature and different levels of humidity. The band intensity at 3350 cm^{-1} (including contribution of both the v1 and v3 normal modes of H_2O) represents full hydration and is defined as 100% (black spectrum). Reducing the humidity in the aerosol leads to less hydrated protein films, whereas the water content of a protein film under dry N_2 is defined as 0% (magenta spectrum). These data form the reference curve in **Fig. 4D** in the main script. **(B)** Second derivative spectra of the same data as in panel A, highlighting the down-shift of the nitrile band when going from 100% to 0% hydration. **(C)** ATR FTIR spectra of the Y3pCNF-*Cp*Fd:*Cp*I complex under 100% N_2 (black), 10% H_2 (red), or 20% O_2 (blue). The amide I and amide II at 1645 cm^{-1} and 1545 cm^{-1} are annotated. The yellow inset is a close-up around 3350 cm^{-1}. These data indicate good stability of the protein film to unspecific hydration changes, although the film is not perfectly stable. The other inset shows an H_2–N_2 difference spectrum in the CO/CN frequency regime of the H-cluster. Negative bands are assigned to H_{ox} while positive bands not only hint at H_{red} and H_{sred} (*) but small traces of reduced states $H_{red'}$ (°) and H_{hyd} (+) as well. **(D)** Tracking the intensity at 3350 cm^{-1} over the time course of the experiment as shown in panel C and discussed in the main script (**Fig. 4**) illustrates a small decrease in hydration level that barely exceeds the experimental variation in absorbance of 5 x 10^{-3}. These changes can be described as not significant.

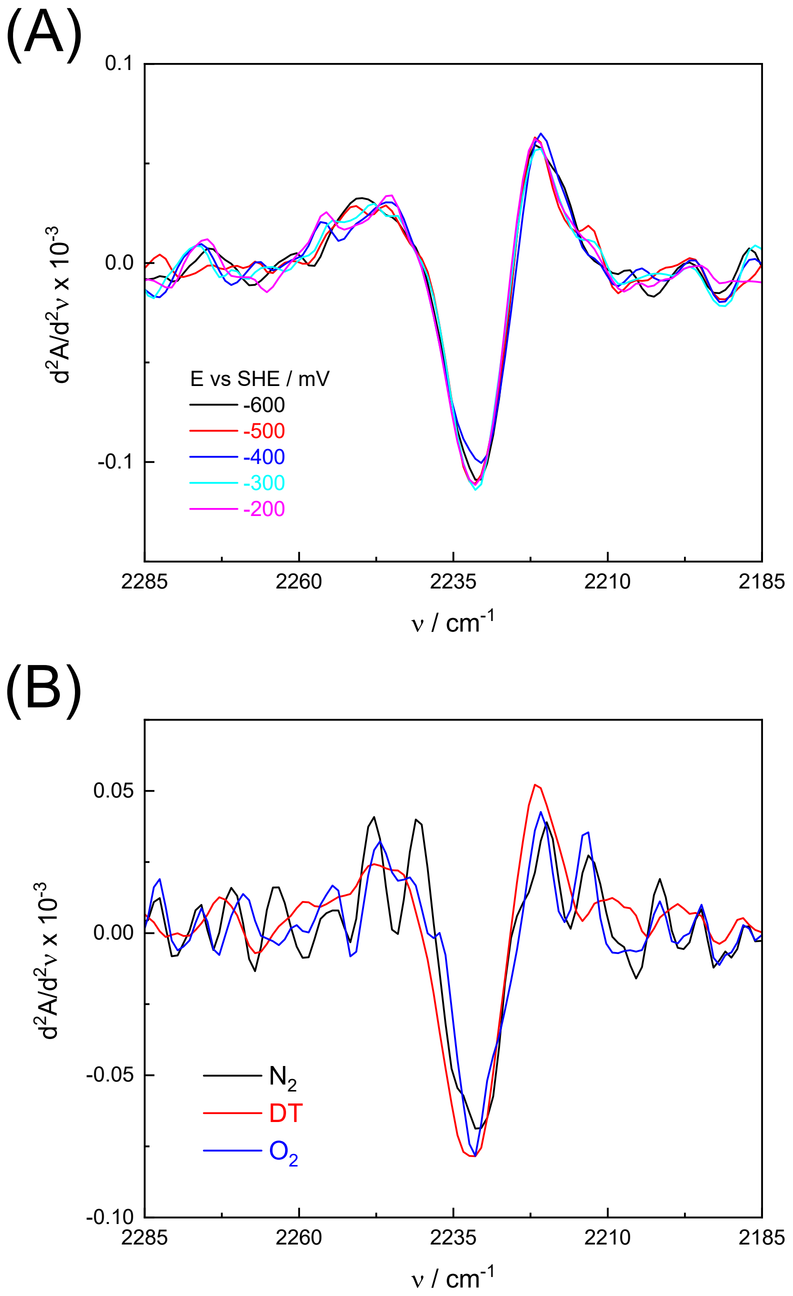

Figure S4. Reduction and oxidation of ferredoxin. (A) Second derivative FTIR spectra of Y3pCNF-*Cp*Fd at electrical potentials between -200 and -600 mV vs. SHE (see (20) for a description of the spectro-electrochemical set-up). These data suggest that the nitrile marker band at 2334 cm^{-1} does not shift upon reduction of Y3pCNF-*Cp*Fd. **(B)** Similar conclusion can be drawn from the reaction of Y3pCNF-*Cp*Fd with dithionite (DT, red trace) and O_2 (blue trace). Compared to the protein under inert conditions (under N_2) no nitrile shifts are observed.

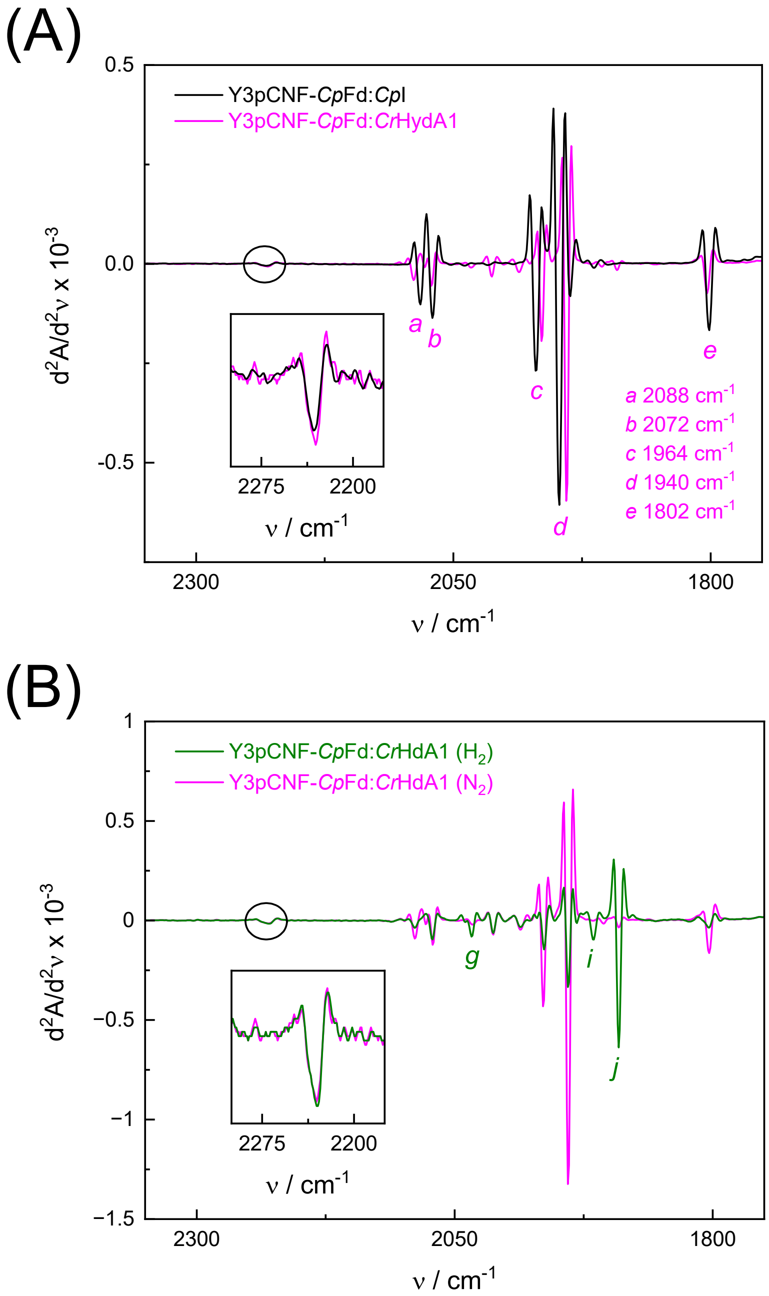

Figure S5. Shift of the nitrile band as a function of redox conditions. (A) Second derivate spectra of Y3pCNF-*Cp*Fd in complex with *Cp*I (black) or *Cr*HydA1 (magenta) under N_2. Bands (a)–(e) are assigned to the H_{ox} state of *Cr*HydA1 and show characteristic shifts relative to *Cp*I (**Tab. S2**). The nitrile band of pCNF is found at ~2230 cm^{-1} in both the Y3pCNF-*Cp*Fd:*Cp*I and Y3pCNF-*Cp*Fd:*Cr*HydA1 complex (inset). **(B)** Second derivate spectra of Y3pCNF-*Cp*Fd in complex with *Cr*HydA1 under N_2 (magenta) or 10% H_2 (dark green). Here, bands (g), (i), and (j) are assigned to the H_{red} state of *Cr*HydA1 (compare **Tab. S2**). We estimate that 70% of the protein was reduced by H_2. Notably, no redox-dependent shifts were observed in the Y3pCNF-*Cp*Fd:*Cr*HydA1 complex (inset).

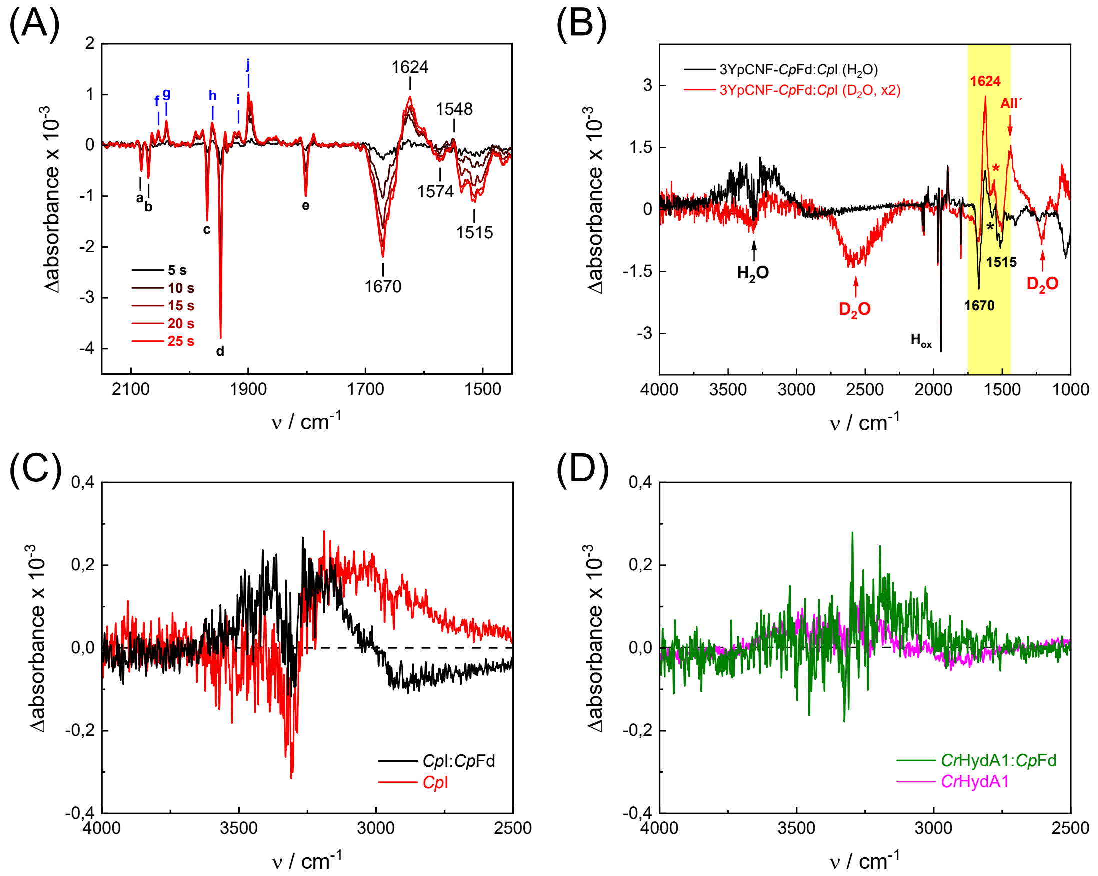

Figure S6. Further evaluation of secondary structural changes. (A) Time series of H_2–N_2 ATR FTIR difference spectra of Y3pCNF-*Cp*Fd:*Cp*I between 5–25 s. Bands above 1750 cm^{-1} are assigned to H-cluster ligands in the H_{ox} state (a)–(e) and H_{red} state (f)–(j). The spectrum includes other reduced states like H_{sred}, $H_{red'}$, and H_{hyd}. See ref. (21) for a description of these H-cluster species. At lower frequencies, the 1670/1624 feature indicates protein structural changes. **(B)** Comparison of ATR FTIR difference spectra for the reaction of the Y3pCNF-*Cp*Fd:*Cp*I complex with H_2 in the presence of H_2O (black spectrum) and D_2O (red spectrum). The deuterated sample shows a small amount of unspecific dehydration (negative "D_2O" bands at 2575 and 1215 cm^{-1}), which result in a slight increase of protein in the beam path, visible from the seemingly tilted baseline at energies >1850 cm^{-1} and the deuterated amide band AII' at 1450 cm^{-1}. Besides these unspecific changes, the 1670/1624 difference feature (yellow mark-up) is unchanged. This excludes an assignment to water (H_2O, D_2O) and agrees with the assignment to amide I secondary structural changes (22). H_2-N_2 difference spectra in the energy regime of the v1 and v3 normal modes of H_2O for *Cp*I samples **(C)** and *Cr*HydA1 samples **(D)**. The data for *Cp*I depict a minor increase of hydration upon reduction with H_2 while the spectral variation in the *Cr*HydA1 samples is barely above noise level. These spectra additionally confirm that the negative band at 1670 cm-1 is unrelated to changes in hydration level and can be assigned to amide I secondary structural changes.

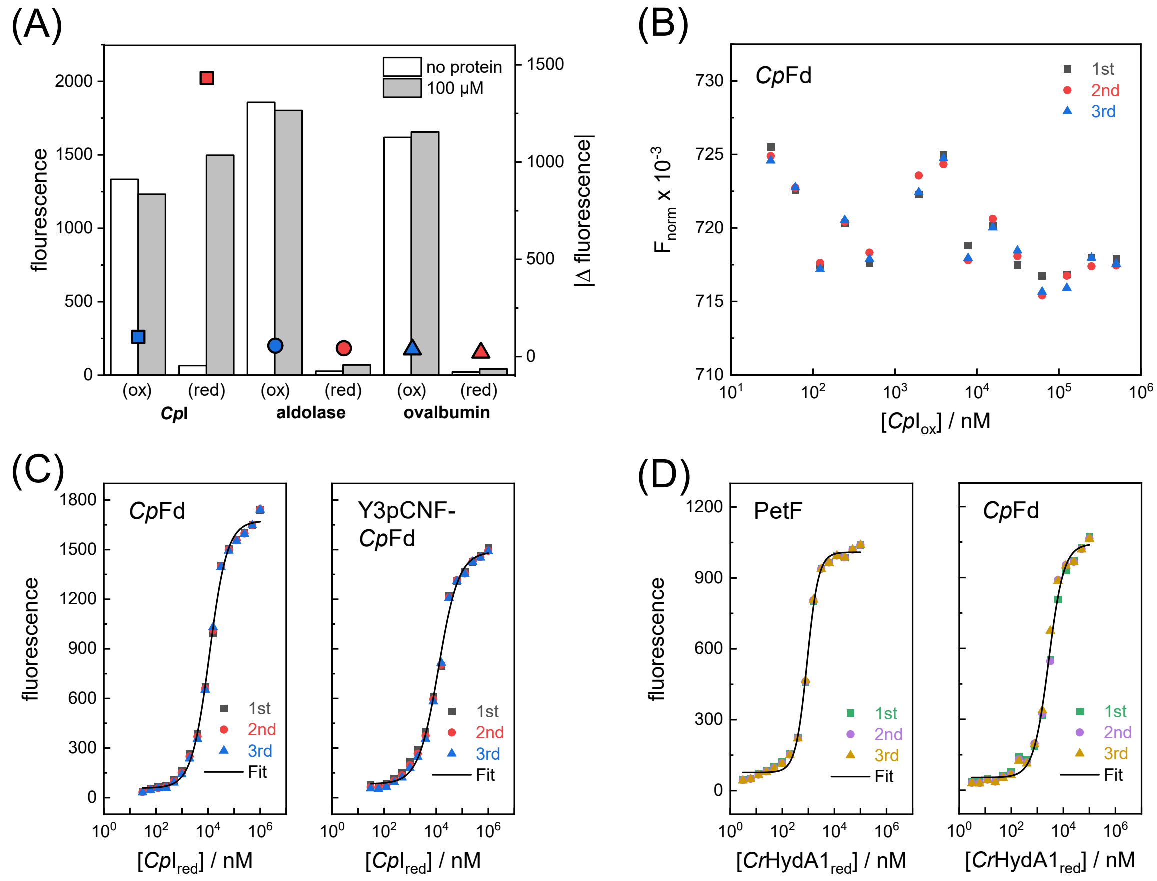

Figure S7. MicroScale Thermophoresis. (A) Fluorescence emission of oxidized (ox) and dithionite-treated (red) Alexa Fluor 647-labeled *Cp*Fd (200 nM) in the presence or absence of *Cp*I or reference proteins aldolase or ovalbumin (each 100 µM). Symbols depict the absolute fluorescence difference "ox-minus-red" highlighting that only *Cp*I can protect Alexa Fluor 647 labeled-*Cp*Fd from bleaching by 2 mM dithionite.(17) **(B)** Concentration-dependent thermophoresis of oxidized Alexa Fluor 647-labeled *Cp*Fd (200 nM) in the presence of *Cp*I (0.03–100 µM) under anaerobic conditions. Fitting the data according to a sigmoid curve is not justified as the data can just as well be fitted by a linear regression. Panels **(C)** depict representative traces of concentration-dependent fluorescence quenching of reduced Alexa Fluor 647-labeled wild-type *Cp*Fd (200 nM) and Y3pCNF-*Cp*Fd (200 nM) in the presence of *Cp*I (0.03–100 µM) and dithionite (2 mM) under anaerobic conditions. From a fit of the data, a K_d of 12.4 ± 0.97 µM was derived for wild-type *Cp*Fd and a similar K_d of 11.3 ± 0.71 µM was obtained for Y3pCNF-*Cp*Fd, suggesting that the mutation does not alter the interaction with *Cp*I. Error bars represent the SD (n = 3). In the FTIR experiments, a *Cp*I/*Cp*Fd ratio of 1:5 was used, which ensures full saturation of *Cp*I but also suggests a significant amount of unbound ferredoxin. The FTIR difference spectra and unaffected by this. Panels **(D)** depict representative traces of concentration-dependent fluorescence quenching of reduced Alexa Fluor 647-labeled wild-type *Cp*Fd (200 nM) or wild-type PetF (200 nM) in the presence of *Cr*HydA1 (0.003–10 µM) and dithionite (2 mM) under anaerobic conditions. From a fit of the data, values K_d = 2.41 ± 0.29 µM (*Cp*Fd) and K_d = 1.24 ± 0.32 µM (PetF) were derived. Error bars represent the SD (n = 4).

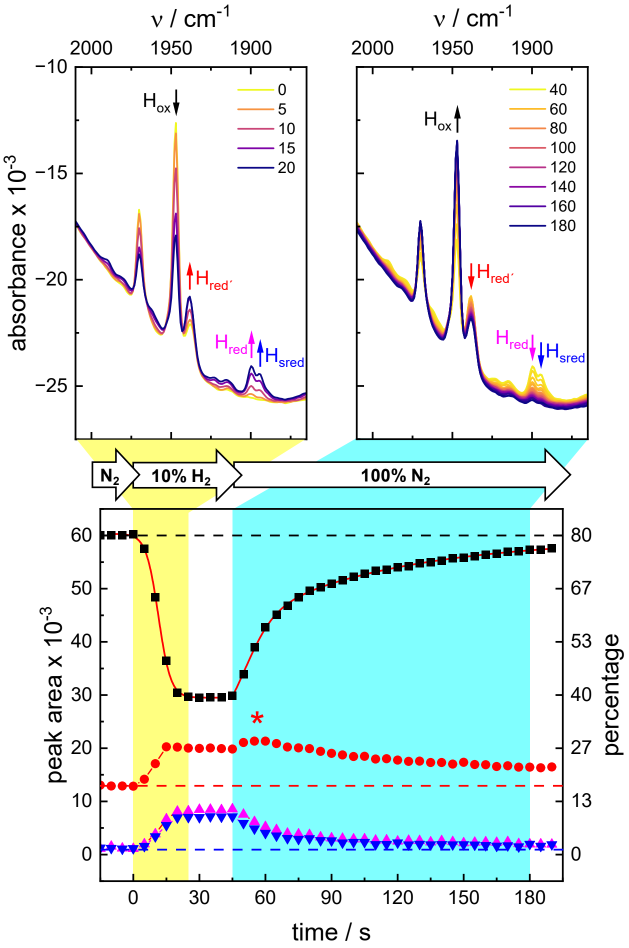

Figure S8. Timeframe of reduction and oxidation. The upper two spectra depict reduction of *Cp*I in the presence of 10% H_2 (left, 0–20 s) and oxidation under 100% N_2 (right, 40–180 s). Characteristic CO bands of H_{ox} (1947 cm^{-1}), $H_{red'}$ (1938 cm^{-1}), H_{red} (1900 cm^{-1}), and H_{sred} (1894 cm^{-1}) are marked. When these bands are fitted, changes in peak area can be followed over time. The graph below shows a rapid decrease of H_{ox} ($k_1 = 2.8 \pm 0.1$ s) and increase in the reduced states in the presence of H_2. When H_2 is removed from the atmosphere, *Cp*I converges back into H_{ox} ($k_{-1} = 51 \pm 1.6$ s) due to proton reduction and "auto-oxidation". The later process is significantly slower but fast enough to adjust for oxidized hydrogenase in the dithionite-free MST experiment.

The temporary increase of $H_{red'}$ (*) when 10% H_2 is switched back to 100% N_2 results from a transfer of electrons from the diiron site (which is reduced in H_{red} and H_{sred}) to the $[4Fe]_H$ cluster (which is reduced in $H_{red'}$). The characteristic offset of $H_{red'}$ (dashed line) shows that *Cp*I is about 80% reduced in the absence of H_2 (the relative accumulation of redox state is giving in percentage on the right Y-axis).

Table S1. Expression yield and purity of purified Fds

**protein**	$\mathbf{A_{390}/A_{280}}$	**yield**<br>(µg/L *E. coli* culture)
*Cp*Fd	0.65	128
Y3pCNF-*Cp*Fd	0.50	26
Y3pCNF-*Cp*Fd *control*	0.36	5

Table S2. H-cluster bands of *Cp*I and *Cr*HydA1 in the H_{ox} and H_{red} states. All frequencies are given in cm^{-1}. Band between 2100–2030 cm^{-1} are assigned to CN^{-} ligands, band between 2030–1890 cm^{-1} are assigned to CO ligands. Below 1890 cm^{-1}, μCO ligands have been assigned.(21)

**state**	**enzyme**	**a**	**b**	**c**	**d**	**e**
H_{ox}	*Cp*I	2082	2070	1970	1947	1800
	*Cr*HydA1	2088	2072	1964	1940	1802
**state**	**enzyme**	**f**	**g**	**h**	**i**	**j**
H_{red}	*Cp*I	2053	2040	1960	1914	1898
	*Cr*HydA1	2072	2033	1960	1915	1892

REFERENCES

1. G. Kuznetsov, *et al.*, Optimizing complex phenotypes through model-guided multiplex genome engineering. *Genome Biol* **18**, 100 (2017).
2. S. J. Miyake-Stoner, *et al.*, Probing Protein Folding Using Site-Specifically Encoded Unnatural Amino Acids as FRET Donors with Tryptophan. *Biochemistry* **48**, 5953–5962 (2009).
3. S. J. Miyake-Stoner, *et al.*, Generating Permissive Site-Specific Unnatural Aminoacyl-tRNA Synthetases. *Biochemistry* **49**, 1667–1677 (2010).
4. J. M. Kuchenreuther, *et al.*, High-Yield Expression of Heterologous [FeFe] Hydrogenases in Escherichia coli. *PLoS One* **5**, e15491 (2010).
5. P. Schönheit, C. Wäscher, R. K. Thauer, A rapid procedure for the purification of ferredoxin from clostridia using polyethyleneimine. *FEBS Lett* **89**, 219–222 (1978).
6. M. Winkler, S. Kuhlgert, M. Hippler, T. Happe, Characterization of the key step for light-driven hydrogen evolution in green algae. *Journal of Biological Chemistry* **284**, 36620–36627 (2009).
7. R. D. Britt, G. Rao, L. Tao, Biosynthesis of the catalytic H-cluster of [FeFe] hydrogenase: the roles of the Fe–S maturase proteins HydE, HydF, and HydG. *Chem Sci* **11**, 10313–10323 (2020).
8. J. Khushvakov, *et al.*, Following Electroenzymatic Hydrogen Production by Rotating Ring–Disk Electrochemistry and Mass Spectrometry. *Angewandte Chemie - International Edition* **60**, 10001–10006 (2021).
9. L. Girbal, *et al.*, Homologous and Heterologous Overexpression in *Clostridium acetobutylicum* and Characterization of Purified Clostridial and Algal Fe-Only Hydrogenases with High Specific Activities. *Appl Environ Microbiol* **71**, 2777–2781 (2005).
10. J. Jumper, *et al.*, Highly accurate protein structure prediction with AlphaFold. *Nature* **596**, 583–589 (2021).
11. D. Kozakov, *et al.*, The ClusPro web server for protein-protein docking. *Nat Protoc* **12**, 255–278 (2017).
12. C. H. Chang, P. W. King, M. L. Ghirardi, K. Kim, Atomic resolution modeling of the ferredoxin:[FeFe] hydrogenase complex from Chlamydomonas reinhardtii. *Biophys J* **93**, 3034–3045 (2007).
13. A. Waterhouse, *et al.*, SWISS-MODEL: homology modelling of protein structures and complexes. *Nucleic Acids Res* **46**, W296–W303 (2018).
14. O. Urquidi, J. Brazard, N. LeMessurier, L. Simine, T. B. M. Adachi, In situ optical spectroscopy of crystallization: One crystal nucleation at a time. *Proceedings of the National Academy of Sciences* **119** (2022).
15. S. T. Stripp, In Situ Infrared Spectroscopy for the Analysis of Gas-processing Metalloenzymes. *ACS Catal* **11**, 7845–7862 (2021).
16. S. T. Stripp, *et al.*, How oxygen attacks [FeFe] hydrogenases from photosynthetic organisms. *Proc Natl Acad Sci U S A* **106**, 17331–17336 (2009).

17. B. Jagilinki, *et al.*, Microscale Thermophoresis (MST) as a Tool to Study Binding Interactions of Oxygen-Sensitive Biohybrids. *Bio Protoc* **14** (2024).
18. M. J. Tucker, R. Oyola, F. Gai, Conformational Distribution of a 14-Residue Peptide in Solution: A Fluorescence Resonance Energy Transfer Study. *J Phys Chem B* **109**, 4788–4795 (2005).
19. L. K. Ries, F. X. Schmid, P. A. M. Schmidpeter, Incorporation of an Unnatural Amino Acid as a Domain-Specific Fluorescence Probe in a Two-Domain Protein. *Biochemistry* **55**, 6739–6742 (2016).
20. S. Mebs, *et al.*, Bridging Hydride at Reduced H-Cluster Species in [FeFe]-Hydrogenases Revealed by Infrared Spectroscopy, Isotope Editing, and Quantum Chemistry. *J Am Chem Soc* **139**, 12157–12160 (2017).
21. J. W. Sidabras, S. T. Stripp, A personal account on 25 years of scientific literature on [FeFe]-hydrogenase. *JBIC Journal of Biological Inorganic Chemistry* **28**, 355–378 (2023).
22. A. Barth, Infrared spectroscopy of proteins. *Biochimica et Biophysica Acta (BBA) - Bioenergetics* **1767**, 1073–1101 (2007).
